# Early-Life Nutrition and Subsequent International Migration: A Prospective Study in Rural Guatemala

**DOI:** 10.1093/jn/nxaa379

**Published:** 2020-12-31

**Authors:** María J Ramírez-Luzuriaga, John F Hoddinott, Reynaldo Martorell, Manuel Ramírez-Zea, Aryeh D Stein

**Affiliations:** Nutrition and Health Science Program, Laney Graduate School, Emory University, Atlanta, GA, USA; Division of Nutritional Sciences and Charles H. Dyson School of Applied Economics and Management, Cornell University, Ithaca, NY, USA; Nutrition and Health Science Program, Laney Graduate School, Emory University, Atlanta, GA, USA; Hubert Department of Global Health, Rollins School of Public Health, Emory University, Atlanta, GA, USA; INCAP Research Center for the Prevention of Chronic Diseases (CIIPEC), Institute of Nutrition of Central America and Panama, Guatemala City, Guatemala; Nutrition and Health Science Program, Laney Graduate School, Emory University, Atlanta, GA, USA; Hubert Department of Global Health, Rollins School of Public Health, Emory University, Atlanta, GA, USA

**Keywords:** child nutrition, growth faltering, Guatemala, international migration, survival analysis

## Abstract

**Background:**

It is generally accepted that migrants are favorably self-selected for labor market skills such as higher schooling and greater cognitive capacity, which are highly correlated with early-life nutrition. However, the influence of early-life nutrition on later-life migration is understudied.

**Objective:**

The objective of this study was to examine prospectively the association between height-for-age *z* scores (HAZ) at 24 mo and subsequent international migration in a cohort of 2392 participants born between 1962 and 1977 in 4 rural villages in eastern Guatemala.

**Methods:**

Information on nutritional status and covariates was collected between 1969 and 1977 and migration status was determined as of 2017 (at ages 40–57 y). We used proportional hazards and logistic regression models to assess whether HAZ was associated with international migration, adjusting for early-life and adult characteristics.

**Results:**

Between 1978 and 2017 there were 297 international migrants (12.4% of the original cohort) during 99,212 person-y of follow-up. In pooled models that were adjusted for early-life characteristics, a 1-SD increase in HAZ was associated with a 19% increase in the risk of international migration (HR: 1.19; 95% CI: 1.02, 1.38). Further adjustment for village characteristics did not alter the estimate substantively (HR: 1.18; 95% CI: 1.02, 1.37), while additional adjustment for schooling attainment attenuated the estimate somewhat (HR: 1.14; 95% CI: 0.98, 1.33). In all models, effect sizes were stronger for men than for women.

**Conclusions:**

Our results indicate that early-life nutrition is positively associated with subsequent international migration.

## Introduction

As of 2020, there were an estimated 272 million international migrants worldwide, a 24% increase since 2010 (1). According to the United Nations, the United States hosts the largest migrant population globally, with nearly 51 million foreign-born individuals (2).

Most immigration to the United States has been from Mexico, even surpassing European migration in the late 1800s and early 1900s (3, 4). In recent years, migration inflows from Mexico to the United States have stabilized and new migration trends have emerged (5). Migration to North America from the Central American countries of El Salvador, Honduras, and Guatemala, the so-called Northern Triangle, has grown markedly, with Mexico serving as a country of transit for migrants in route to the United States (5, 6).

Migration is a complex and dynamic process driven by the interplay of a set of social, economic, environmental, and political factors in sending and receiving countries. Political instability, insecurity, violence, poverty, unemployment, and natural disasters serve as “push factors” in sending countries, whereas “pull factors,” acting from receiving countries, include better income and job prospects, higher standards of living, strong social and family ties, and better educational systems (7, 8).

Due to the complexity of migration patterns, no single theory explaining the causes of migration has been widely accepted. The neoclassical theory assumes that individuals migrate to maximize their income (8). The human capital framework enriches the neoclassical approach by considering sociodemographic characteristics as important predictors of migration (e.g., age, gender, skills, education, marital status, occupation, preferences, and expectations) (8). The new economics of migration theory further recognizes the influence of family, households, and communities in the decision-making process (9).

The evidence on predictors of international migration has remained mixed and inconclusive. The most commonly studied characteristics have included age, gender, education, socioeconomic, professional, and marital status (10–12). A common limitation in these studies has been their reliance on aggregate and cross-sectional data. Moreover, these studies have focused on characteristics measured in adulthood and after the fact, potentially overestimating the influence of such characteristics if they are correlated with often unobserved early-life factors (7, 12–14). It is generally accepted that economic migrants (those who move from one region to another, or across borders to benefit from greater economic opportunities) tend to have on average a more favorable set of skills for labor market success than similar individuals who choose to stay in their place of origin (11). While migrants are favorably self-selected for labor market skills such as higher schooling and greater cognitive capacity, which are highly correlated with early-life nutritional status (15), the influence of early life nutrition on later life migration has not been assessed.

Growth faltering in early life, defined as a failure to reach one's linear growth potential, is a marker of malnutrition and a strong correlate of human, social, and economic capital over the life course (15–18). Growth faltering occurs in contexts of poverty and low education and results from a combination of inadequate feeding, childcare practices, and infections (19). Conversely, better growth in the first 2 y of life has been associated with school attainment, better job prospects, and higher standards of living (15, 17).

In this study, we aimed to examine prospectively the association between growth faltering in early life and subsequent international migration in a well-established prospective cohort of Guatemalan adults. A previous study conducted among the nonmigrants of this population documented positive associations between HAZ at 24 mo and human capital outcomes (15). We consider international migration to be a human capital outcome and thus we hypothesized that linear growth in childhood will be positively associated with international migration.

## Methods

### Study population

This study was based on a prospective cohort of 2392 individuals born between 1962 and 1977 in 4 rural communities in eastern Guatemala who were enrolled as children in a community-randomized food supplementation trial conducted from 1969 to 1977 (20). The purpose of the trial was to assess the effect of improved nutrition on the mental development of preschool children (21). Participants have been followed up on several occasions, including 1988–1989, 2002–2004, and 2017–2019, when the cohort had mean ages of 18, 33, and 47 y, respectively.

### Variable characterization

#### Assessment of international migration and age at migration

In each follow-up wave (1988–1989, 2002–2004, and 2017–2019), a census was conducted in each village. As part of this work, the survey teams ascertained the current location (original study village, nearby village, Guatemala City, elsewhere in Guatemala, living outside Guatemala) of each living participant in the original cohort. The approximate date of international migration and country of destination were obtained from the migrant's families and trusted intermediaries residing in the original study villages. For each participant, we created a variable, “international migrant,” that was given a value of 1 when the respondent was identified as having ever lived outside Guatemala, or 0 if not.

The exact age at migration was known for 131 (44%) participants who migrated internationally. In the remaining 166 (56%) participants who were international migrants with unknown age at migration, we imputed age at migration as follows. In participants identified as international migrants in 2002–2004 who did not participate in the 1988–1989 follow-up, age at migration was imputed as the midpoint between 1978 and 2001 (16.2%, *n* = 48); in participants identified as international migrants in 2002–2004 who did participate in the 1988–1989 follow-up, the age was imputed to the midpoint between 1988 and 2001 (16.8%, *n* = 50). In participants first identified as international migrants in 2017–2019, the age was imputed to the midpoint between 2002 and 2017 (22.9%, *n* = 68).

#### Characterization of linear growth

During the 1969–1977 trial, child length was measured 15 days after birth, every 3 mo through age 24 mo, every 6 mo through age 60 mo, and then at 72 and 84 mo using length boards. Body measurements were taken by trained and standardized anthropometrists using standardized protocols (22, 23). To convert lengths to standing heights, we subtracted 1.0 cm from length measures obtained at 24 mo and younger (24). We converted heights into height/length-for-age *z* scores (HAZ) using the WHO growth standards (25, 26). HAZ is a continuous measure of attained linear growth and values below −2 SD are used to characterize stunting. We focused on HAZ at 24 mo as a predictor of international migration. We had direct measures on HAZ at age 24 mo for 899 children. Values of HAZ were imputed for children without a measure at 24 mo of age but with measures at other ages using the available measure closest in age to 24 mo (*n* = 960). The robustness of this approach has been assessed elsewhere (15).

### Potentially confounding variables

#### Early-life characteristics

Family characteristics of study participants, including the years of schooling of both parents and mother's height and age at childbirth, were extracted from files created during the 1969–1977 nutritional trial. Ownership of household assets and housing characteristics of study participants were ascertained by interview in village censuses conducted in 1967 and 1975. A combined index score was created using principal component analysis to capture the socioeconomic characteristics of study participants in early childhood. The 1967 index score was used for participants born before January 1, 1971, and the 1975 index score was used for participants born on or after that date (27). From village developmental histories, proxy measures were constructed for village characteristics at various points in time, which may have influenced out-migration from the study villages. These variables were schooling availability and quality at ages 8 and 13 y (i.e., the primary school student: teacher ratio and whether the primary school was a permanent cement-block structure) and labor market participation at ages 13 and 18 y (i.e., nearby cement industry boom and good access roads) (28).

#### Attained schooling

To assess schooling, we used information collected in 2002–2004 on completed years of formal and informal education, and for participants with missing information in that follow-up (∼17%) we used data collected in the 2017–2019 follow-up. Informal education data consists of literacy programs available in Guatemala for adults who wished to complete primary or secondary school.

### Inclusion and exclusion

**FIGURE 1:**
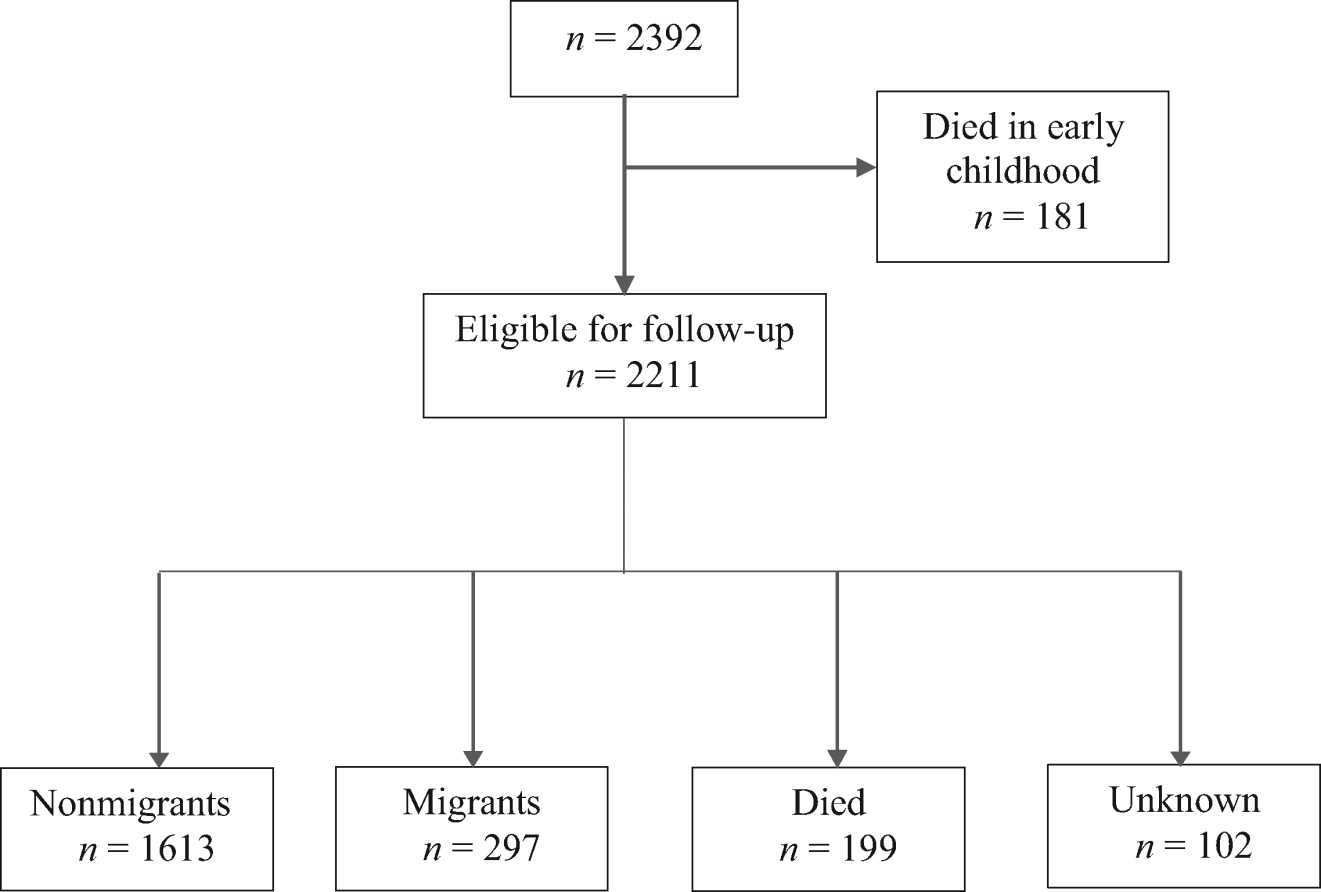
Flowchart of study participants by 2017–2019 follow-up.

From the 2392 individuals enrolled in the 1969–1977 nutritional trial, those who had died in early childhood (before 1978) were excluded from the analysis (*n* = 181), resulting in 2211 participants who comprise our main analytical sample. In some analyses, we further excluded individuals who had died by 2017–2019 (*n* = 199) or were lost to follow-up (*n* = 102) resulting in 1910 participants (**Figure 1**).

### Statistical analysis

To examine the association of linear growth with international migration we fitted models with HAZ at 24 mo as the primary predictor. We used Cox proportional hazards regression models to examine if HAZ at 24 mo was associated with time to international migration. Participants contributed person-years from the date the trial ended to the date international migration occurred, or the date of response to the last follow-up wave, whichever came first. We used the participant's age at each time point as the time scale.

We defined 4 models a priori, with progressive adjustment for confounders. In model 1, we adjusted for year of birth to control for period or cohort effects and village dummy variables to control for all time-invariant characteristics of the study villages. Because the proportion of participants with siblings in the trial was high, we adjusted the SEs for within-family correlations. In model 2, we additionally adjusted for the years of schooling of both parents, mother's height and age at childbirth, and socioeconomic status in early childhood. These early-life characteristics are proxies for maternal and paternal socioeconomic conditions that could have influenced international migration.

In model 3, we additionally adjusted for village characteristics that may have encouraged out-migration from the study villages (i.e., proxy measures of schooling availability and quality and labor market variables). Last, because schooling is an important predictor of human capital that may have increased local labor market opportunities and economic resources, facilitating the occurrence of international migration, we additionally adjusted for the participant's completed years of schooling (model 4). We present pooled and sex-stratified results. Pooled models additionally controlled for sex.

For covariates with missing values, we used multiple imputation techniques, which produce unbiased estimates under the assumption of missing at random. The proportion of missing information was 15.9% for HAZ at 24 mo, 25.5% for mother's height, 8.9% for mother's schooling, 14.3% for father's schooling, 2.1% for mother's age at childbirth, and 26.2% for attained schooling.

In sensitivity analyses, we additionally modeled the association of linear growth and international migration using logistic regression models adjusting for the same set of covariates previously described and using multiple imputation techniques for missing covariates. The proportion of missing information was 13.9% for HAZ at 24 mo, 22.6% for mother's height, 5.4% for mother's schooling, 10.2% for father's schooling, 1.1% for maternal age at childbirth, and 18.6% for attained schooling.

All statistical tests were 2-sided and considered statistically significant at *P* < 0.05. All analyses were conducted using STATA 16.

## Results

In the period between 1978 and 2017, we observed 297 cases of international migration during 99,212 person-y of follow-up. The majority of international migrants were men (63%). Age at migration ranged from 14 to 46 y, and most international migration occurred to the United States (**Supplemental Table 1**). Mean HAZ scores at 24 mo, socioeconomic status in childhood, and years of schooling were all higher among international migrants (**Table 1**).

**TABLE 1:** Descriptive statistics of study population by follow-up status in 2017–2019^1^

Variable	Nonmigrants (*n* = 1613)	Migrants (*n* = 297)	Dead (*n* = 199)	Lost to follow-up (*n* = 102)
Gender, male	1613 (47.5)	297 (63.3)	199 (62.3)	102 (52.9)
Year of birth	1613 (1970 ± 4)	297 (1970 ± 4)	199 (1970 ± 5)	102 (1969 ± 3)
HAZ at 24 mo	1398 (−3.18 ± 1.1)	245 (−2.94 ± 1.0)	168 (−3.34 ± 1.2)	48 (−3.38 ± 1.1)
Maternal age at childbirth, y	1602 (27.1 ± 7.1)	287 (26.4 ± 6.9)	194 (27.0 ± 7.0)	82 (24.7 ± 7.6)
Maternal schooling, y	1549 (1.3 ± 1.6)	257 (1.6 ± 1.8)	178 (1.3 ± 1.9)	30 (0.5 ± 1.1)
Paternal schooling, y	1472 (1.8 ± 2.2)	243 (1.8 ± 2.3)	157 (1.3 ± 2.1)	23 (1.0 ± 1.7)
Maternal height, cm	1263 (148.5 ± 5.2)	215 (149.4 ± 5.2)	140 (148.5 ± 5.4)	29 (148.1 ± 4.9)
Childhood SES tertile, %				
Poorest	1613 (35.1)	297 (23.9)	199 (35.7)	102 (27.4)
Middle	1613 (33.2)	297 (35.0)	199 (31.2)	102 (47.1)
Wealthiest	1613 (31.6)	297 (41.1)	199 (33.2)	102 (25.5)
Attained schooling, y	1443 (4.9 ± 3.6)	110 (6.2 ± 3.7)	67 (4.5 ± 2.8)	11 (3.4 ± 3.5)
Village school permanent structure				
Age 7 y, %	1613 (48.6)	297 (54.5)	199 (46.2)	102 (48.0)
Age 13 y, %	1613 (82.5)	297 (85.2)	199 (79.4)	102 (84.3)
Village student:teacher ratio^2^				
Age 7 y	1613 (40.0 ± 8.3)	297 (39.8 ± 8.1)	199 (39.3 ± 8.4)	102 (40.3 ± 8.2)
Age 13 y	1613 (36.1 ± 7.5)	297 (35.4 ± 7.0)	199 (36.0 ± 7.3)	102 (36.1 ± 7.1)
Village cement industry boom				
Age 13 y, %	1613 (48.5)	297 (58.9)	199 (60.3)	102 (42.2)
Age 18 y, %	1613 (56.0)	297 (68.3)	199 (72.4)	102 (45.1)
Village good access road				
Age 13 y, %	1613 (31.7)	297 (23.6)	199 (24.6)	102 (34.3)
Age 18 y, %	1613 (43.9)	297 (41.7)	199 (40.2)	102 (50.1)

1 Values are total *n* (% with variable) or total *n* (mean ± SD of variable) unless otherwise indicated. Percentages of missing information are 15.9% for HAZ at 24 mo, 2.1% for maternal age at childbirth, 8.9% for maternal schooling, 14.3% for paternal schooling, 25.5% for maternal height, and 26.0% for attained schooling. HAZ, height-for-age *z* scores; SES, socioeconomic status.

2 Number of students divided by the number of teachers in village school when individual was 7 and 13 y old.

In pooled models, a 1-SD increase in HAZ was associated with 24% increase in the risk of international migration (HR: 1.24; 95% CI: 1.09, 1.40) (**Table 2**). In models that adjusted for early-life factors that did not include village characteristics, a 1-SD increase in HAZ was associated with a 19% increase in the risk of international migration (HR: 1.19; 95% CI: 1.02, 1.38). The further adjustment for village characteristics resulted in an estimated 18% increase in the risk of international migration (HR: 1.18; 95% CI: 1.02, 1.37). With additional adjustment for schooling, the estimate was attenuated (HR: 1.14; 95% CI: 0.98, 1.33).

**TABLE 2:** Cox proportional estimates of the association between HAZ at 24 mo and international migration^1^

	Pooled (*n* = 2211)	Women (*n* = 1079)	Men (*n* = 1132)
Model 1^2^	1.24 (1.09, 1.40)	1.26 (1.01, 1.56)	1.23 (1.06, 1.43)
Model 2^3^	1.19 (1.02, 1.38)	1.17 (0.92, 1.49)	1.21 (1.03, 1.44)
Model 3^4^	1.18 (1.02, 1.37)	1.16 (0.92, 1.47)	1.21 (1.02, 1.43)
Model 4^5^	1.14 (0.98, 1.33)	1.13 (0.88, 1.44)	1.16 (0.98, 1.40)
Cases of migration, *n*	297	109	188
Person-y, *n*	83,103	40,506	42,597

1 Estimates are HRs (95% CIs) unless otherwise indicated. HAZ, height-for-age z-scores.

2 Adjusted for fixed effects of birth village and birth year.

3 Additionally adjusted for years of schooling of the mother and father, mother's age at childbirth, mother's height, and socioeconomic status in early childhood.

4 Additionally adjusted for permanent primary structure at ages 7 and 13 y, student:teacher ratio at ages 7 and 13 y, cement boom at age 13, and good access to roads at ages 13 and 18 y.

5 Additionally adjusted for the participant's completed years of schooling. In all models, confidence intervals account for clustering at the mother level and pooled models adjusted for sex. For missing covariates, we used multiple imputation techniques.

Estimates using logistic regression models were consistent with those determined by using Cox proportional hazard regression models. In pooled models that adjusted for early-life characteristics, a 1-SD increase in the HAZ was associated with a 20% increase in the likelihood of international migration (OR: 1.20; 95% CI: 1.02, 1.41). The further adjustment for village characteristics resulted in an estimated 19% increase in the likelihood of international migration (OR: 1.19; 95% CI: 1.02, 1.40) and the estimate was attenuated with additional adjustment for schooling attainment (OR: 1.15; 95% CI: 0.97, 1.36) (**Table 3**). In all models, effect sizes were stronger for men than for women.

**TABLE 3:** Logistic regression estimates of the association between HAZ at 24 mo and international migration^1^

	Pooled (*n* = 1910)	Women (*n* = 956)	Men (*n* = 954)
Model 1^2^	1.24 (1.08, 1.43)	1.25 (0.98, 1.59)	1.24 (1.05, 1.47)
Model 2^3^	1.20 (1.02, 1.41)	1.18 (0.90, 1.52)	1.23 (1.02, 1.47)
Model 3^4^	1.19 (1.02, 1.40)	1.17 (0.90, 1.52)	1.23 (1.02, 1.47)
Model 4^5^	1.15 (0.97, 1.36)	1.14 (0.85, 1.49)	1.18 (0.97, 1.42)

1 Estimates are ORs (95% CIs). HAZ, height-for-age *z* scores.

2 Adjusted for fixed effects of birth village and birth year.

3 Additionally adjusted for years of schooling of the mother and father, mother's age at childbirth, mother's height, and socioeconomic status in early childhood.

4 Additionally adjusted for permanent primary structure at ages 7 and 13 y, student-teacher ratio at ages 7 and 13 y, cement boom at age 13, and good access to roads at ages 13 and 18 y.

5 Additionally adjusted for the participant's completed years of schooling. In all models, CIs account for clustering at the mother level and pooled models adjusted for sex. For missing covariates, we used multiple imputation techniques.

## Discussion

To our knowledge, this study provides new and unique evidence on predictors of international migration. Previous studies have used aggregate data and have focused on characteristics measured in adulthood and after international migration has occurred. Using individual-level data from a longitudinal cohort in Guatemala with nearly 50 y of follow-up, we linked HAZ at 24 mo (a continuous measure of linear growth) with international migration, adjusting for potential confounders.

Our study findings show that better early-life nutrition is associated with international migration, and the association is attenuated when schooling attainment is included in the model. We did not examine explicitly the pathways through which early-life growth influences international migration, mostly because we lacked the data in migrants to undertake such an approach. However, our findings suggest that one of the pathways through which early-life growth associates with subsequent international migration may operate via schooling. Growth failure in early childhood is a marker of malnutrition and other early-life deprivations occurring during critical periods in brain development (29). Consequently, growth failure in the first 2 y of life has been linked to adverse long-term consequences for schooling attainment and other human capital indicators (15, 17). Attaining higher grades of schooling may create new opportunities such as higher-return labor market activities, and thus more available resources for international migration. It is well documented that those who migrate are not the poorest, partly because migrants need the financial resources to support their travel (7, 10, 30). This proposed mechanism is well aligned with previous studies among the nonmigrants in this population, which have documented that a 1-SD increase in HAZ was associated with 0.78 more grades of schooling and lower probability of being poor (15). However, the existing evidence on the influence of early-life nutrition on various aspects of development and human capital in this population have excluded international migrants from the analyses, which might have led to the sample being selective and the estimates understated.

Our study also shows that the association between HAZ and international migration was robust to confounding factors (i.e., socioeconomic status, gender), which have already been widely identified as important predictors of international migration. We found that international migrants came from families with higher socioeconomic status in childhood than nonmigrants, which is well aligned with previous studies conducted in similar populations (12). Moreover, in agreement with previous literature, men showed higher rates of outmigration than women. A comparative analysis assessing differences in patterns of male and female migration in 5 Latin American countries (Mexico, Costa Rica, Puerto Rico, the Dominican Republic, and Nicaragua) found that in more patriarchal societies, female heads of households were less likely to migrate than male heads of households, whereas in more matriarchal settings the ratio of female to male migration was much higher (31). Guatemala has, for the most part, a traditional patriarchal system of family and gender relations, particularly in rural villages where most international migrants originate.

Moreover, our findings support the healthy migrant hypothesis, which is based on the notion that those who migrate are healthier than the population they leave behind (32). This hypothesis is often used to explain an epidemiological phenomenon commonly referred to as the “Hispanic paradox” where despite having a more disadvantaged risk profile, the Hispanic immigrant population in the United Stated experiences better health outcomes than the general US population (33).

There are important social and political factors that help contextualize our study results. The INCAP (Institute of Nutrition of Central America and Panama) longitudinal study took place during a civil war that lasted almost 40 y (1960–1996). The civil war was mostly targeted toward Mayan populations who were subject to political persecution (34). The region where the original trial was conducted was predominantly a ladino area (mixed Spanish and indigenous descent) with a very low Mayan population density. Participants in the original study were also ladino; as a result, political persecution was not a major driver of migration in our study population. During the early 1980s, emigration in Guatemala increased significantly due to a combination of economic and political factors that resulted from the civil war (35). In the postwar era, increased migration from Guatemala to the United States continued once migrant communities in the United States were established and in response to the country's socioeconomic problems, natural disasters, and increasing social violence influenced by drug traffickers, organized crime, and gangs (35). Migration, in most cases, has been a survival strategy for Guatemalans who have the economic resources to finance the trip to the United States. In 2015, the country was listed among the top remittance-receiving countries in the world, with the United States–Guatemala corridor accounting for 5.8 billion of 6.4 billion dollars received that year (36).

There are some limitations to this study. Age at migration was imputed in more than half of the international migrants (∼56%), potentially affecting the precision of estimates. However, estimates using logistic regression models (which do not require age at migration) were very similar to those obtained using Cox proportional hazard models. Also, there is a possibility of nondifferential misclassification of international migration, especially because study follow-ups were widely spread, and some instances of short-term migration could have been underidentified (false negatives). However, given the close relationship that the research team has built with the study participants and the study villages in almost 50 y of collaboration, (which includes frequent updates of information of members of the cohort), we believe this factor had minimal impact on the estimates since it is unlikely that a case went unidentified. An additional limitation is that we did not control for unobserved characteristics in childhood and adolescence that could potentially be associated with childhood linear growth and are likely to have an influence on international migration.

This study also has strengths. To our knowledge, our analysis is unique for 2 reasons. First, we used individual level rather than aggregate data. Second, we assessed information collected during infancy and early childhood, which distinguishes this study from many others that lack such information. In addition, the use of survival analysis coupled with multiple imputation techniques ensured efficient use of available data improving precision around the estimates without introducing bias.

## Supplementary Material

nxaa379_Supplemental_File

## Notes

Supported by the Bill and Melinda Gates Foundation (OPP1164115).

Author disclosures: The authors report no conflicts of interest. ADS is a member of the Journal's Editorial Board.

Supplemental Table 1 is available from the “Supplementary data” link in the online posting of the article and from the same link in the online table of contents at https://academic.oup.com/jn/.
