# Supplementary material for: Early-Life Nutrition and Subsequent International Migration: A Prospective Study in Rural Guatemala
Source: J Nutr. 2020 Dec 31;151(3):716–21. doi: 10.1093/jn/nxaa379 (PMC7948204; doi:10.1093/jn/nxaa379)
Supplement: nxaa379_Supplemental_File [file nxaa379_supplemental_file.docx]

**Early-life nutrition and subsequent international migration: A prospective study in rural**

**Guatemala**

María J. Ramírez-Luzuriaga, Online Supplementary Material

**Supplemental Table 1.** International migrants by country of destination and sex in the Nutrition Supplementation Trial Longitudinal Cohort.

|  | Females (*n* =109) | |  | Males (*n* =188) | |
| --- | --- | --- | --- | --- | --- |
|  | n | % |  | n | % |
| Country of destination |  |  |  |  |  |
| United States | 82 | 75.2 |  | 150 | 79.8 |
| Mexico | 2 | 1.83 |  | 2 | 1.06 |
| Central America | 2 | 1.83 |  | 1 | 0.53 |
| Canada | 0 | 0 |  | 2 | 1.06 |
| Europe | 5 | 4.58 |  | 1 | 0.53 |
| Unknown | 18 | 16.5 |  | 32 | 17.0 |
